# Biofunctionalization of zinc oxide nanowires for DNA sensory applications

**DOI:** 10.1186/1556-276X-6-511

**Published:** 2011-08-25

**Authors:** Raphael Niepelt, Ulrich C Schröder, Jana Sommerfeld, Irma Slowik, Bettina Rudolph, Robert Möller, Barbara Seise, Andrea Csaki, Wolfgang Fritzsche, Carsten Ronning

**Affiliations:** 1 Institute of Solid State Physics, Friedrich-Schiller-Universität, Max-Wien-Platz 1, 07743 Jena, Germany; 2 Institute of Photonic Technology (IPHT), PO Box 100239, 07702 Jena, Germany

**Keywords:** nanowires, zinc oxide, functionalization, fluorescence microscopy, sensor technology

## Abstract

We report on the biofunctionalization of zinc oxide nanowires for the attachment of DNA target molecules on the nanowire surface. With the organosilane glycidyloxypropyltrimethoxysilane acting as a bifunctional linker, amino-modified capture molecule oligonucleotides have been immobilized on the nanowire surface. The dye-marked DNA molecules were detected via fluorescence microscopy, and our results reveal a successful attachment of DNA capture molecules onto the nanowire surface. The electrical field effect induced by the negatively charged attached DNA molecules should be able to control the electrical properties of the nanowires and gives way to a ZnO nanowire-based biosensing device.

## Background

Semiconductor nanowires have gained large interest as building blocks for low-cost, highly sensitive biosensors [1,2]. Due to the quasi-one-dimensional geometry of the nanowires and the large surface-to-volume ratio, surface-induced effects play a significant role on the electrical properties of nanowire-based devices. Even a single molecule attached to the nanowire surface is able to change the electrical properties of the nanowire considerably. Therefore, a functionalization of the nanowire surface gives way to highly sensitive sensors that can be arranged in a very dense assembly, owing to their nano-sized dimension. However, both the surface modification and the nanowire device arrangement have to be taken care of in order to prepare a complete nanowire-based DNA sensor.

During the last decade, there have been several studies concentrating on the development of Si-based nanowire sensors [1-3]. Si nanowires are complementary metal oxide semiconductor (CMOS) compatible and biocompatible, and techniques for the functionalization of Si are well understood. Although it is not CMOS compatible, ZnO is very stable under ambient conditions and compared to silicon especially its surface is stable under oxygen-rich conditions. The stability of ZnO nanostructures under physiological conditions, which is important for biosensing applications, depends on the crystal quality of the nanostructures but can be assumed as sufficient for structures grown by thermal evaporation methods [4-6]. The material is also biocompatible, non-toxic, and easy available. Nanostructures made of ZnO are easy and reliable to produce in a wide manner of different forms and structures [7,8]. The DNA capture molecule immobilization with organosilanes has been successfully shown by Corso and co-workers on planar ZnO surfaces [9], where the biomolecule layer was used to act as an acoustic wave sensor. With the use of ZnO nanowires instead of planar films, the metal oxide itself can be used to build an electrically working biosensor. Biosensors based on ZnO nanostructures have been reported, using both electrostatic adsorption as well as organosilane modification with subsequent covalent binding for immobilization [10-13]. Various enzymes have been immobilized using electrostatic adsorption in order to realize highly selective bioassays [11-13]. Recently, the biofunctionalization of ZnO nanowires with biotin for streptavidin detection has been shown using organosilanes with aldehyde functionality that bind the amino group of biotin [10]. The nanowire-based protein sensors could detect streptavidin binding down to a concentration of 2.5 nmol, underlining the capability of ZnO nanowires for the electrical detection of biomolecules. Hereby the nanowire has the character of an active channel in a field effect transistor whose conductivity is governed by the field effect induced by the assembly of charged target molecules on the semiconductor surface.

In this study, we will extend the successful biofunctionalization of ZnO nanowire surfaces to DNA using the organosilane glycidyloxypropyltrimethoxysilane (GOPS) [14-16]. DNA molecules are negatively charged and therefore applicable for the electrical detection with biofunctionalized nanowires. In contrast to the detection of streptavidin in [10], where the current increases when streptavidin is attached, the current through the intrinsically *n*-type ZnO nanowire should decrease as the negatively charged DNA target molecules induce a depletion zone inside the nanowire.

## Experimental

The ZnO nanowires have been grown via vapor-liquid-solid mechanism in a horizontal tube furnace [17] at a pressure of 150 mbar with a growth temperature of 1,050°C. The nanowires evolve in a typical spaghetti-like structure with wire diameters around 100 nm and wire lengths up to some tens of micrometers. To remove adsorbed species from the nanowires and supply a clean and reactive ZnO surface, the samples have been oxygen-plasma-treated for 30 min at 5 Pa oxygen pressure with a plasma energy of 50 W. As this treatment is known to enhance the dissociative adsorption of water on the ZnO surface [18], the samples have been stored in a cabinet dryer at 100°C for at least 5 min before the silane treatment to remove water residues from the surface.

**Figure 1:**
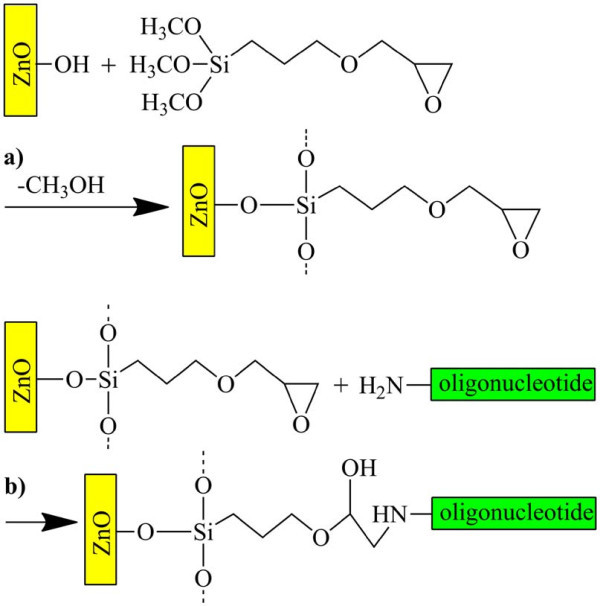
**Schematic view of the biofunctionalization of ZnO**. In a toluene environment, (**a**) the bifunctional GOPS molecule is attached to the ZnO nanowire surface. In a second step, (**b**) the oligonucleotide is linked to the epoxy group of the GOPS [6].

Subsequent silanization took place in 10 mM GOPS in a water-free toluene solution (for details on chemicals resource, see Additional file 1). The silanization mechanism is displayed in Figure 1a. The samples have been incubated stirring constantly for at least 6 h at 70°C. The samples have been rinsed three times subsequently with pure toluene for 5 min in each case and then dried with compressed air.

The 5'-amino-modified oligonucleotides were dissolved in 5 × PBS buffer (pH 7.4) with a concentration of 10 μmol for the biofunctionalization. The single-stranded DNA sequences of the applied oligonucleotides consisting of 27 bases are shown in Table 1. The fluorescence markers that are linked to the 3'-end and enable the detection of the molecules with fluorescence microscopy are also listed in Table 1. The DNA solution was pipetted onto the nanowire samples and incubated overnight under wet conditions to immobilize the nucleotides on the GOPS surface via the 5'-amino linker (see Figure 1b). Afterward, the samples were rinsed for 10 min in 15 mmol Tris-HCl buffer (pH 8) in order to get rid of unbound DNA and then dried with compressed air.

**Table 1:** DNA sequence and fluorescence markers of the oligonucleotides used for the ZnO nanowire biofunctionalization

5'-Modification	Sequence from 5'-3'	3'-Modification
C6-aminolink	TCT TAG TTC CTC GTG TAC GAC TTT TTT	FITC
C6-aminolink	TCT TAG TTC CTC GTG TAC GAC TTT TTT	Cy3

To ensure covalent binding following the scheme in Figure 1 takes place, control samples have been prepared both leaving out the GOPS silanization step and with DNA that does not have an amino linker. The biofunctionalized nanowires were mechanically transferred to silicon or glass substrates by a gentle pressure imprint of the growth substrate.

The as-grown nanowires have been characterized with a JEOL JSM-6490 scanning electron microscope. Subsequently to the biofunctionalization, the samples were investigated with a Zeiss Axio Imager 2 fluorescence microscopy setup. Fluorescence microscopy is known as an adequate tool for the analysis of DNA monolayers on thin film surfaces as well as on nanowires [9,19]. The microscope makes use of a filter cube to detect marker-specific fluorescence. The samples marked with cyanine 3 (Cy3) dyes turned out to be more photo stable than the fluorescein isothiocyanate (FITC)-marked samples. Thus, we will present here mainly Cy3-marked samples.

## Results and discussion

**Figure 2:**
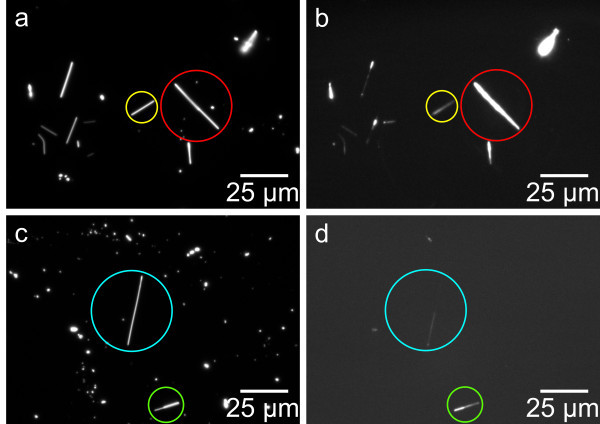
**Dark field (a and c) and fluorescence (b and d) images of biofunctionalized ZnO nanowires**. Nanowires in upper row have been modified with Cy3-marked DNA and nanowires in bottom row with FITC-marked DNA. Note that the nanowires marked with the red and yellow circles in (b) show different fluorescence intensities. The nanowire marked blue in (d) only shows fluorescence at one side; the nanowire marked green shows higher fluorescence intensity at the side that is appearing darker in the corresponding dark field image.

**Figure 3:**
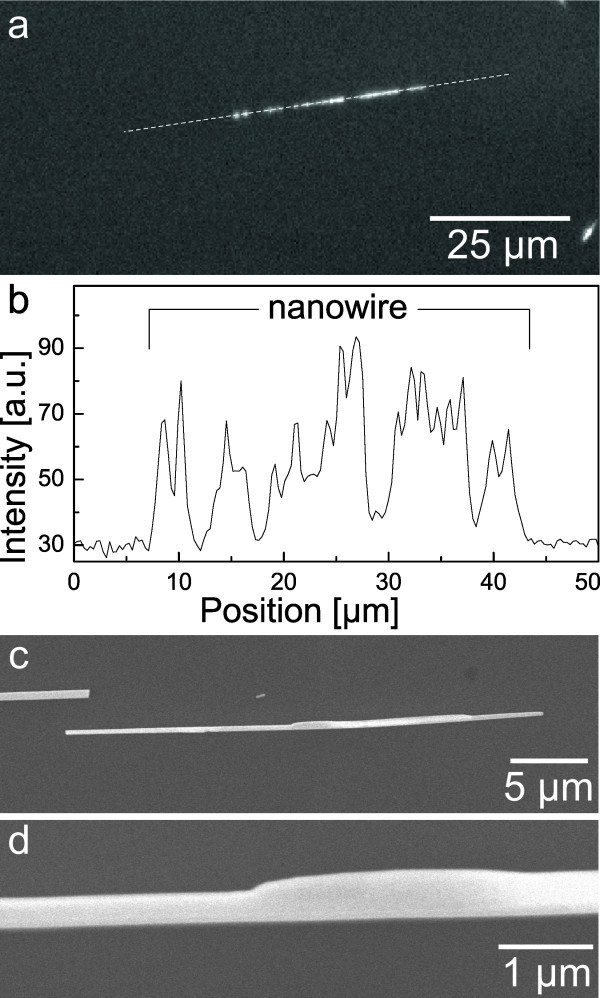
**Nanowire intensity fluctuations and thickness variations**. In (**a**), a fluorescence image of a biofunctionalized nanowire is shown. In (**b**), the intensity plot along the dotted line is shown. The intensity value is fluctuating along the wire axis and even disappearing at two points. Images (**c**) and (**d**) show SEM images of a typical nanowire used in this study. With the larger magnification in (d), the nanowire thickness variation is clearly visible.

Dark field and fluorescence images of biofunctionalized ZnO nanowires are shown in Figure 2a,b,c,d. One can clearly see that every nanowire that can be identified in the dark field image is showing a fluorescence signal. However, the luminescence intensities differ extremely for the investigated wires. This can be explained by the fact of different diameters of the nanowires and thus different nanowire surface areas. The intensity of the emitted light is also appearing non-uniformly along the nanowire axis, as clearly visible on the wire highlighted in Figure 2d. These intensity fluctuations might be owing to only fragmentary DNA coverage or variations of the nanowire thickness. In Figure 3b, the fluorescence intensity along the nanowire axis (dotted line) in Figure 3a is shown. It is clearly visible that the fluorescence intensity is not only fluctuating but even disappearing in some regions of the wire. This leads to the assumption that the functionalized nanowire might not be completely covered with DNA.

In Figure 3c,d, detailed scanning electron microscope (SEM) pictures of a typical ZnO wire used in this study are shown. One can see that the wire itself has a non-uniform thickness, varying up to a factor of 2. The detailed surface morphology of the functionalized wires is not clearly resolvable within an SEM; however, a uniform coverage of the nanowire is noticed. A respective SEM image can be found in the supplementary information (Additional file 1). Thus, we can also trace back a major distribution to the nanowire intensity fluctuations to the thickness variations along the wire axis. To confirm this estimation, we also functionalized a single crystalline ZnO bulk sample and investigated the DNA surface coverage. The sample is showing a homogeneous fluorescence of the functionalized area (not shown here, see Additional file 1), underlining that the GOPS treatment is applicable for the successful accumulation of DNA monolayer to ZnO surfaces.

The observed thickness variations might result from furnace-related instabilities of the growth conditions. With a scaled-up and improved setup, it is possible to overcome this issue easily [20].

However, it is possible that due to the uneven nanowire surface of the samples, a uniform coverage of the nanowire with DNA molecules might be hampered. Nanowires sticking together during the silanization process could also lead to a non-uniform DNA coverage. Additionally, damages of the DNA layer during the imprint could result in the same layer inhomogeneity. Nanowires grown from a seed array [20] could be used to avoid non-uniform coverage due to shadowing effects and facilitate a non-destructive nanowire imprint process. Therefore, the intensity fluctuations of the biofunctionalized nanowires can be explained by the interplay of nanowire thickness variations and non-homogeneous surface coverage. Recourse to either can be solved using a more advanced nanowire growth setup than the one utilized here.

As ZnO nanostructures are known to show a green defect luminescence that can be dramatically enhanced when the nanowires are coated [21], one has to check if the wires themselves might cause a significant luminescence signal that could be interpreted falsely as fluorescence. Therefore, all samples have been examined with different band pass fluorescence filters. All samples only showed fluorescence at wavelengths referable to the specific dye used for the biofunctionalization; a broader defect luminescence was not detected.

**Figure 4:**
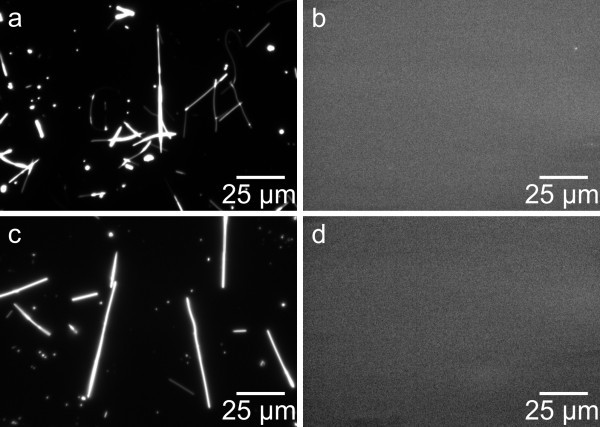
**Reference experiments with untreated nanowires**. In (**a**) and (**b**), the dark field and fluorescence image of an unmodified nanowire sample are shown. In (**c**) and (**d**), the dark field and fluorescence image of a silanized but not biofunctionalized nanowire sample are shown. In both cases, the fluorescence measurement does not show any sign of distracting fluorescence.

Additionally, necessary reference experiments have been performed with untreated ZnO wires as well as nanowires, which were silanized but not biofunctionalized (with DNA). Figure 4 confirms the absence of autofluorescence or luminescence of the nanowires.

It is also known that ZnO nanostructures act fluorescence enhancing themselves through affecting the self-quenching properties of fluorophores [22]. This could lead to fluorescent wires on top of a dye-covered substrate. As mentioned already above, the wires in our study were first functionalized and then subsequently transferred to a clean and dye-free substrate. Thus, fluorescence enhancement of substrate-bonded fluorescence markers can also be excluded as the origin of the observed fluorescence.

**Figure 5:**
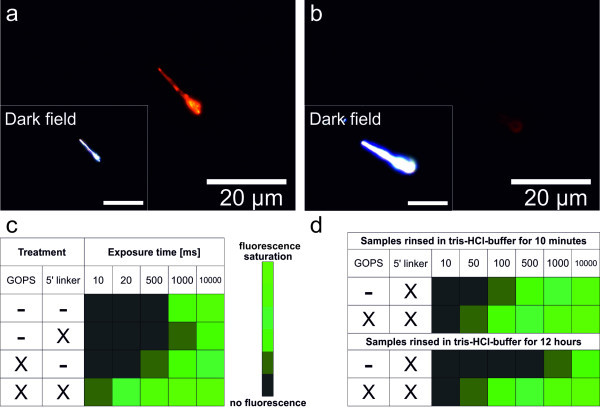
**Control experiments to confirm a covalent bonding of DNA**. In (**a**), a typical fluorescence image of a silanized and biofunctionalized nanowire is shown. In (**b**), an image of a nanowire is shown that was treated the same way, but leaving out the silanization step and using DNA without a 5'-amino linker. In (**c**), the fluorescence intensities of various control samples are compared. In (**d**), a comparison of a sample with GOPS and a sample without GOPS rinsed in Tris-HCl buffer solution for different periods of time is presented.

Upon having clarified that the observed fluorescence is caused by DNA attached to the ZnO nanowires, the question regarding the character and stability of this attachment still remains. To investigate these issues, several control samples have been prepared. In Figure 5, a typical fluorescence image of a silanized and biofunctionalized nanowire (a) is compared to an image of a nanowire that was treated the same way, but leaving out the silanization step and using DNA without a 5'-amino linker (b). One can see that the observed luminescence appears to be orders of magnitude weaker in the latter case. In Figure 5c, the fluorescence intensities of various control samples are compared. To quantify the intensities of different fluorescent samples, all signals have been integrated for different lengths of time and the resulting apparent magnitudes have been classified into six levels of fluorescence intensity between no fluorescence at all and fluorescence saturation. It turns out that the fluorescence is most pronounced in the case of GOPS treatment and DNA with amino linker. All the other control samples show significant less fluorescence, indicating that the strongest binding of the DNA to the nanowire is only achieved with both coupling groups present (epoxy group at GOPS and amino group at DNA) in order to form a covalent binding.

In Figure 5d, a comparison of a sample with GOPS and a sample without GOPS is presented. Both samples were biofunctionalized with DNA with amino linker. The samples have been rinsed in Tris-HCl buffer solution for 10 min and then measured. As expected, the silanized sample is showing the stronger fluorescence. However, after rinsing both samples for another 12 h, the fluorescence of the sample silanized with GOPS and then functionalized has not dropped, whereas the fluorescence of the control sample is less present than before. This also indicates a comparatively stable, presumably covalent bond between the DNA and the nanowire following the reaction scheme in Figure 1. In conclusion, the stable biofunctionalization of ZnO nanowires has been clearly demonstrated. The bifunctional linker GOPS was used to immobilize a monolayer of DNA capture molecules on the ZnO nanowires. The successful functionalization could be verified with fluorescence microscopy. Variations of the nanowire thickness result in fluorescence variations along the nanowire axes. The functionalized wires may be used as building blocks for electrically driven DNA target molecule detection.

## Competing interests

The authors declare that they have no competing interests.

## Authors' contributions

US and IS synthesized and characterized the nanowires and conducted the fluorescence microscopy measurements. RN assisted with the nanowire synthesis and drafted the manuscript. JS participated in the fluorescence microscopy measurements and helped to draft the manuscript. BR and BS carried out the biofunctionalization and assisted with the fluorescence microscopy measurements. RM and AC participated in the design of the study and its coordination. CR and WF participated in the design of the study and helped to draft the manuscript. All authors read and approved the final manuscript.

## Supplementary Material

Additional file 1 **Supplementary information**. Detailed information about the chemicals used in this study, fluorescence images of biofunctionalized bulk crystals, and SEM images of a biofunctionalized nanowire. Click here for file
