# Supplementary material for: Biofunctionalization of zinc oxide nanowires for DNA sensory applications
Source: Nanoscale Res Lett. 2011 Aug 25;6(1):511. doi: 10.1186/1556-276X-6-511 (PMC3212050; doi:10.1186/1556-276X-6-511)
Supplement: Additional file 1 — Supplementary information. Detailed information about the chemicals used in this study, fluorescence images of biofunctionalized bulk crystals, and SEM images of a biofunctionalized nanowire. [file 1556-276X-6-511-S1.PDF]

Biofunctionalization of zinc oxide nanowires for DNA sensory applications

Raphael Niepelt[1*§], Ulrich C. Schröder[1*], Jana Sommerfeld[1*], Irma Slowik[1], Bettina Rudolph[2], Robert Möller[2], Barbara Seise[2], Andrea Csaki[2], Wolfgang Fritzsche[2], Carsten Ronning[1§]

[1]Institute of Solid State Physics, Friedrich-Schiller-Universität, Max-Wien-Platz 1, 07743 Jena, Germany

[2] Institute of Photonic Technology (IPHT), PO Box 100239, 07702 Jena, Germany

*These authors contributed equally to this work

[§]Corresponding author

Supplementary information: ZnO (99.99%) powder for nanowire growth was obtained from Alfa Aesar. The GOPS was produced by Sigma-Aldrich. The oligonucleotides used in this paper were synthesized by Eurofins MWG Opera. All other chemicals used have been supplied by Roth.

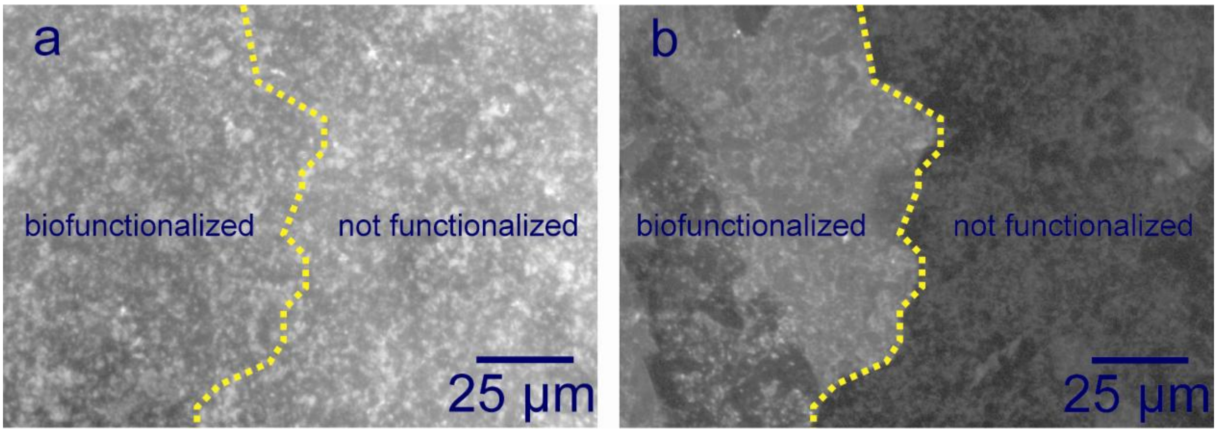

Figure SI1. Bright field (a) and fluorescence (b) image of a partially biofunctionalized ZnO bulk crystal surface. The sample is showing a homogeneous fluorescence of the biofunctionalized area, underlining that the GOPS treatment is applicable for the successful accumulation of DNA monolayer to ZnO surfaces.

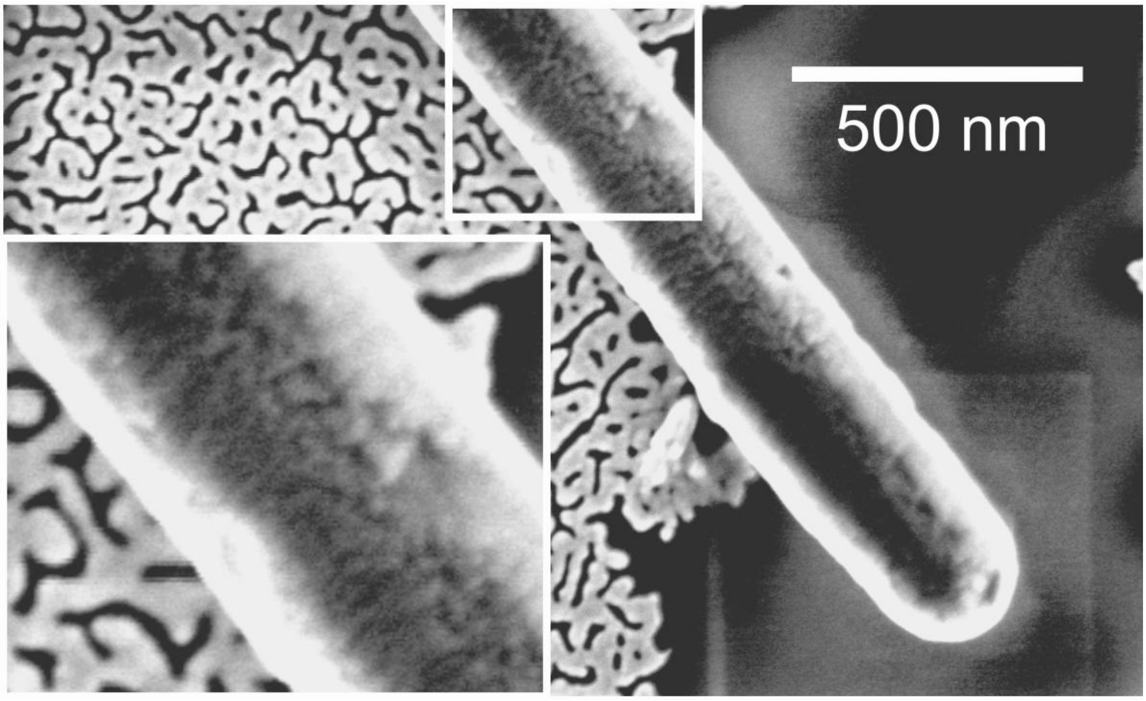

Figure SI2. FE-SEM study of a biofunctionalized ZnO nanowire. The inset shows an enlarged view of the white rectangle in the top middle of the picture. To avoid charging interferences, the nanowire was imprinted to a substrate covered with a thin gold layer. The picture shows a rough but homogeneous coverage of the nanowire surface. Single biomolecules cannot be resolved with the FE-SEM.
